# *Varroa destructor* weakens the external immunity of western honey bees by impairing melittin production

**DOI:** 10.1038/s41598-025-13440-2

**Published:** 2025-08-20

**Authors:** Michelina Pusceddu, Simon Tragust, Panagiotis Theodorou, Irene Ciabattini Bolla, Jorge Sánchez Navarro, Francesco Corrias, Alessandro Atzei, Alberto Angioni, Ignazio Floris, Alberto Satta

**Affiliations:** 1 Department of Agricultural Sciences, Section of Plant Pathology and Entomology, University of Sassari, Sassari, Italy; 2 National Biodiversity Future Center (NBFC), Palermo, Italy; 3 General Zoology, Institute of Biology, Martin Luther University Halle-Wittenberg, Halle (Saale), Germany; 4 Department of Life and Environmental Sciences, University of Cagliari, Cagliari, Italy

**Keywords:** *Apis mellifera*, Mite, Ectoparasite, Bee venom, Self-grooming, Allogrooming, Ecology, Zoology, Diseases

## Abstract

Social insects employ venom as an external immune defence against pathogens and parasites. Like other Hymenopterans, the venom gland of honey bee (*Apis mellifera*) serves as a reservoir of antimicrobial substances, primarily melittin. This study investigated the presence and origin of venom on honey bee body that could act as an external immune defence in honey bee workers infested by the ectoparasite mite, *Varroa destructor*. Using a multi-step approach, we first confirmed the presence of venom on bees’ bodies using melittin as a marker. We then examined how grooming could facilitate the distribution of venom on the bee’s body through behavioural observations. Further assays were used to compare melittin levels on the bodies of *Varroa*-free and *Varroa*-infested workers and assess the effects of bee-venom on mite activity. Our findings confirmed the occurrence of “venom bathing” in *A. mellifera*, excluding social components or environmental contamination, with bees likely coating their bodies with antimicrobial substances through self-grooming. Our results further suggest that infested bees spread higher amount of venom on their bodies compared to uninfested bees, and bee-venom significantly reduced mite activity, suggesting that the venom functions as an external defence. However, *Varroa* negatively impacted melittin production. Our study reveals a previously unknown negative effect of *V. destructor*: impairment of honey bees’ external immune defence through reduced melittin production.

**Supplementary Information:**

The online version contains supplementary material available at 10.1038/s41598-025-13440-2.

## Introduction

Collaboration among individuals in social insect colonies leads to important benefits in brood care, foraging, antipredator defence, and other group survival activities ^1^. However, the close and frequent interactions among genetically homogeneous nestmates, the large amount of food stored in the nest, and the relatively stable conditions in the nest-environment may also promote the spread of parasites and pathogens within the colony ^2,3^. To counteract the risk of disease contraction and transmission within their societies, apart from their body’ innate immune system, social insects possess a diversity of physiological, behavioural and organizational adaptations, collectively often called “social immunity” ^4–10^. Many adaptations against parasites and pathogens act in the environment of a social insect society. They can thus also be conceptualized as external immune defence mechanism, i.e. traits acting outside an individual and either improving the protection from pathogens and parasites or manipulating the composition of the microbial community in favour of the individual ^11^ and thus directly or indirectly benefiting the individual and the society it lives in. For example, ants and honey bees incorporate tree resins with antimicrobial properties in their nests, thus modifying the microbial community of the nest environment ^12–15^. In the case of ants, resin collection seems to be a mechanism implemented to prevent disease ^16^, whereas in the case of honey bees, this behaviour can also have curative purposes, as suggested for the *Ascosphaera apis* fungus ^17^ and the *V. destructor* mite ^18–20^. In addition to environment-derived bioactive substances (e.g., resins), self-produced substances, especially from exocrine glands such as the venom gland, can play a key role in external immunity ^21,22^. In many species of aculeate Hymenoptera, the sting apparatus and venom originally evolved as tools to kill prey, but later became a means of defence against predators, mainly vertebrates ^23^. However, the venom of aculeate Hymenoptera is also a source of antimicrobial substances ^24,25^, suggesting its potential use as an immune defence trait. The adaptive role of venom to sanitize oneself, the nest, other nest members and food is well known in ants reviewed in ^21,26^, which can apply and spread their antimicrobial venom during grooming behaviour e.g., ^27^. Whether honey bees, or bees in general, also use their venom for sanitary purposes as ants do is less clear.

Like ant venoms, bee venoms, especially in honey bees, are pharmacologically active products that consist of a complex mix of biogenic amines, proteins, peptides, phospholipids, sugars, and volatile components ^28–31^. Among species in the genus *Apis*, venom production can be influenced by seasonality ^32^, age and/or caste ^33–36^, and body size ^37^. However, even in species with similar body size, differences in venom amount and composition often occur. For instance, although the Eastern honey bee *A. cerana* produces only half as much venom as the Western honey bee *A. mellifera*, its venom contains a higher proportion of the peptide melittin ^37^. Melittin is the main component of honey bee venom, accounting for 50% of the venom’s dry weight ^38,39^. Melittin has antiseptic ^24^, strong antiviral ^40^, antifungal and antimicrobial properties ^41^. Apart from melittin, antimicrobial activity has also been suggested for other bee venom peptides such as apamin and Mast Cell Degranulation ^42^.

In honey bees, the possible use of venom as an external immune defence trait in a social immunity context was (to our knowledge) first hypothesized by Baracchi and Turillazzi ^43^, who found traces of venom compounds on the body of adult workers and on the surface of honeycombs. Later, the same authors found that, although melittin was present in the venom of both open-nesting and cavity-nesting bees, it was only detectable on the cuticle and combs of the cavity nesters ^44^, that nesting ecology and the environment shape a specie s’ deposition of venom, potentially due to different associated disease pressures. Finally, with respect to the overall amount of venom peptides both in the venom and on the cuticle, the same authors ^44^ suggested that *A. cerana* performs a higher level of *“venom bathing”* compared to *A. mellifera,* which is also in accordance with a higher rate of grooming activity shown by *A. cerana* compared to *A. mellifera* and grooming being one of the main mechanisms of resistance against parasitic mites in the eastern honey bee ^45^. A relationship between grooming and venom is also suggested through the observation that, for both bee species, venom peptides are completely absent on the cuticle of freshly emerged bees, which probably cannot produce the venom yet, as well as adult drones ^43,44^.

**Fig. 1:**
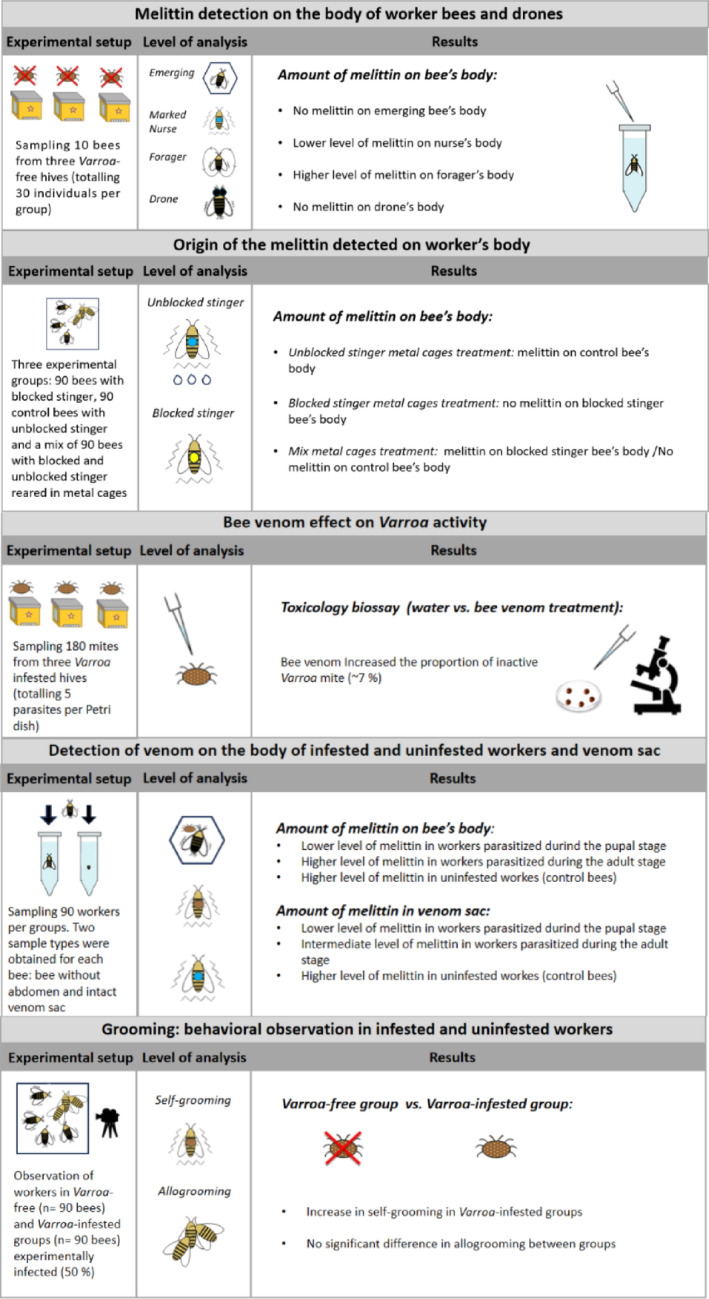
Graphical abstract.

Considering that previous research suggested the role of venom in honey bees is well beyond the classical defence activity against predators ^44^, our study investigated the presence and origin of venom on the honey bee body (*venom bathing behaviour*) and whether it can act as an external immune defence trait upon challenge by *V. destructor* together with venom effects on this parasite’s activity. We first performed chemical analyses to confirm the presence of venom on the bodies of nurse and forager workers, using melittin as a marker. To exclude the possibility that the detected melittin originated from environmental contamination within the nest, we also quantified the amount of melittin on the bodies of drones and freshly emerged workers. Additionally, to ascertain that the melittin detected on the workers’ bodies originated from venom produced by the bees themselves and smeared on the cuticle through grooming, we quantified the amount of venom on bees with either blocked or unblocked stingers. These bees were kept together in groups or separately. We then investigated if venom transfer to the body surface of honey bees was influenced by the presence of *Varroa* mites. To do this, we quantified the amount of venom (using melittin as a marker) on the bodies of mite infested (both at the pupal and adult stage) and uninfested workers for comparison. To ensure that bees in the three experimental groups had an equal supply of venom and could thus equally utilize this defence mechanism, we also quantified the amount of melittin inside their venom sacs. In addition, since previous studies have suggested that grooming in *A. mellifera* is less effective than in *Apis cerana* in counteracting the mite ^45–50^, but it may play an additional role in relation to the spread of venom, we determined the frequency and efficiency of self-grooming and allogrooming through behavioural observations comparing infested and uninfested groups of workers. Finally, through toxicological laboratory assays, we evaluated whether a biologically relevant dose of bee venom could induce lethality or suppress activity in the ectoparasite *V. destructor*. All experiments are summarised in Fig. 1.

## Materials and methods

### Chemical analysis

#### Standards and reagents

We purchased melittin at the certified analytical standard (purity ≥ 85%) by Sigma Aldrich (Milan, Italy), acetonitrile (ACN) at the LC/MS grade (Sigma Aldrich, Milan, Italy), formic acid at the reagent grade (> 95%, Honeywell, Sigma Aldrich, Milan, Aldrich), and HNO_3_ (67–69%) as an ultra-pure grade solvent (Romil Spa, Cambridge, England). MilliQ water (18.25 MΩ × cm) was obtained from an integrated Millipore purification system (MilliQ, Merck, Milan, Italy). A melittin stock solution (1000 µg Ml ^– 1^) was prepared by solubilization in a 0.1% aqueous formic acid solution and stored at 4 °C until use. The working solution (200 µg mL ^– 1^) was freshly prepared each day by diluting the stock solution with a 0.1% aqueous formic acid solution. The five-point calibration curve was obtained by diluting the working solution with a 0.1% formic acid aqueous solution at concentrations of 0.1, 0.25, 0.50, 1, and 1.5 µg mL ^– 1^.

#### Extraction of melittin from bees and the venom sac

Individual bees (without abdomen) and venom from glands were placed into 1.5 mL microcentrifuge tubes for mellitin analysis. Venom was recovered manually from the glands of the sting apparatus ^51^. Bees were frozen at − 20 °C and the sting apparatus was removed using sterilised precision tweezers. Venom was recovered from the glands with a capillary tube and stored in 1.5 mL microcentrifuge tubes before analysis. Microcentrifuge tubes with individual bees (without abdomen) or venom were added to 1 mL of 0.1% aqueous formic acid solution and mixed for 1 min in vortex and for 15 min in a rotary shaker (Reax Top, Heidolph, Germany). The extracting solution was transferred to 1.8 vials and analysed by LC-MS/MS.

#### LC–MS/MS analysis

Analytical determinations of the solution from extracted bees were performed using an Agilent 1290 Infinity II UHPLC coupled to an Agilent 6470 Triple Quad LC-MS/MS mass detector paired with a MassHunter ChemStation. The column was a ZORBAX Eclipse Plus C18 (2.1 × 150 mm, 1.8 µm). A binary gradient, water + 0.1% formic acid (A) and ACN + 0.1% formic acid (B) was set as follows: T = 0 at 95% A, T = 4.30 min at 15% A, T = 5.80 min at 15% A, T = 7.70 min at 95% A, and 2 min post-run at 95%. The flow rate was 0.2 mL min^-1^, with 5 μL sample volume injected in positive mode. The mass detector gas and the sheet gas were set at 300 °C and 250 °C, with flow rates 5 L min ^– 1^ and 11 L min ^– 1^, respectively. The nebulizer was held at 30 psi and the capillary voltage was 4000 V. Melittin has a molecular weight (MW) of 2646.46 Da. Ionization in the positive ESI mode produces a stable MH + 4 ion with a MW of 712.44 (Table S1). Analyses were carried out in dynamic MRM mode (Table S2).

#### Analytical method validation

To validate our analytical method, we followed the SANTE guidelines by evaluating linearity, selectivity, precision, limits of quantification of the method (LOQ), accuracy in terms of recovery, uncertainty, and matrix effect ^52^. Six control bees were fortified by depositing an aliquot of solution of the analytical standard at 1 µg mL ^– 1^, left to rest for 24 h, and extracted as reported above. Each sample belonged to an independent experiment. Six solutions of the analytical standard at 0.1 µg mL ^– 1^ (LOQ) and 1 µg mL ^– 1^ (10 × LOQ) were analysed within one day to verify their repeatability (RSDr, intraday). Reproducibility (RSDwR) was calculated by analysing two analytical standard solutions over six days. Recovery results were analysed using matrix control standard calibration curves. The matrix effect was evaluated by comparing the analytical response of melittin in water + 0.1% formic acid with solutions prepared with extracts from unfortified control (blank) bees. Linearity was assessed by analysing standard calibration curves performed on five different days and was considered acceptable when the coefficient of determination was greater than 0.990. Selectivity was assessed by comparing the extracts of unfortified control bees with those spiked with the standard. The absence of chromatographic peaks at melittin retention times was a criterion for the selectivity of the confirmatory method. The expanded measurement uncertainty (U), a quantitative parameter of the reliability of the analytical method, was calculated by multiplying the combined uncertainty (u′) by a coverage factor k = 2, to obtain a 95% confidence level, using the following equation ^53^:

$$u^{\prime } = \sqrt {u^{\prime } } (bias)2 + u^{\prime } (precision)2;\,U = k\, \times \,u^{\prime }$$

The instrumental LOQ was calculated as the lowest fortification level that meets the method identification and performance criteria regarding recovery and precision.

### Experimental apiary

The study was performed from April 2023 to November 2023 in an experimental apiary of the Department of Agricultural Sciences of the University of Sassari located in Ottava, Sardinia (Italy; latitude 40°46′23″, longitude 8°29′34″). The apiary consisted of twelve colonies set up in May 2022 with *Apis mellifera ligustica* queens sharing a homogeneous genetic profile (sister queens) maintained in ten-frame Dadant-Blatt hives. During this period, the colonies were monitored every two weeks to check for the presence of the queen and food stores and to evaluate the sanitary status of the bees (disease symptoms and varroosis). Before selecting the colonies to be used in the experiment, the *V. destructor* infestation level (%) of each colony was assessed following standard method ^54^. This method involves collecting a sample of approximately 300 adult bees per colony from three different frames, which are then sacrificed at  − 20 °C. Afterwards, these bees are bathed in a hydroalcoholic solution to facilitate *Varroa* separation. The percentage of infestation is calculated by counting the number of mites relative to the total number of adult bees in the sample. The infested colonies were used as a source of *V. destructor* mites and infested adult workers (cell infestation), whereas the uninfested ones (infestation level < 1%) were used as a source of uninfested adult workers.

### Detection of melittin on the body of adult worker bees and drones

We sampled ten individuals per category from three different colonies: freshly emerged bees, nurses, foragers, and drones (totalling 30 individuals per category-group). Each sampled bee was immediately placed individually inside a 1.5 mL microcentrifuge tube, sacrificed on dry ice in field, and stored at –20 °C without its abdomen until chemical analyses were performed by following the procedure for individual bees described above. The abdomen was detached to prevent contamination of the collected bees’ bodies during subsequent manipulations necessary for chemical analyses.

Freshly emerged bees were directly sampled upon their emergence. To collect nurse bees, freshly emerged bees were marked on the abdomen with a non-toxic colour upon emergence, returned immediately to their original hives and, seven days later, sampled from the hives. Forager bees with pollen load were captured at the hive entrance. Drones were sampled from inside the colonies.

### Origin of the melittin detected on the workers’ bodies

To establish the origin of melittin on the bee’s body surface, we quantified the amount of melittin present on the bodies of bees belonging to the following experimental groups: (1) 90 bees with blocked stingers, (2) a mix of 90 bees with blocked (45) and unblocked stingers (45), and 3) 90 bees with unblocked stingers (control group). The stinger was blocked using a stick to apply a droplet of non-toxic glue onto the tip of the abdomen. All bees were marked on the abdomen with a non-toxic colour identifying the treatment group. To obtain uninfested adult workers of the same age, were collected combs with brood ready to emerge from three *Varroa*-free colonies and kept them for 14 h in an incubator at + 35 °C ^18^. To exclude the family effect, experimental groups were replicated in three independent metal cages (10 cm × 10 cm × 5 cm), each containing a mix of 30 emerging bees each from three *Varroa*-free colonies (ten bees per colony per cage), on the same day ^18^. The larger inner side of the cages was covered by a sheet of bee-wax (9 cm × 9 cm), whereas the opposite side was closed by a glass window (10 cm × 10 cm). All cages were kept in an incubator (+ 31.5 °C, 70% R.H., dark) with a 50% (w/v) sucrose solution administered ad libitum with a graduated syringe ^55^. On the seventh day after emerging, all bees were sacrificed on dry ice and stored without the abdomen at –20 °C until chemical analyses.

### Detection of melittin on the body of infested and uninfested workers and in the venom sac

To determine if the amount of venom produced, using melittin as a proxy, varied quantitatively on the body surface of *Varroa-*infested and uninfested bees, we set up the following experimental groups: (1) 90 workers parasitized by *Varroa* during the adult stage only, (2) 90 workers parasitized during the pupal stage, and (3) 90 uninfested workers (control). To obtain uninfested workers of the same age from the *Varroa*-free colonies, we used the method described above ^18^. To obtain adult workers parasitized during the adult stage (dispersal phase), female mites were sampled from brood cells capped in the preceding 15 h, as described by Nazzi et al. ^56^. To assess infestation success, mite movement on adult bees was observed on the first day of the experiment, and mite mortality was recorded for each cage on the final day as a double control. To obtain adult workers of the same age parasitized during the pupal stage (cell infestation), we collected and kept under observation for about 8 h frames with brood ready to emerge from three *Varroa*-infested colonies ^20^. Each emerging bee was checked for the presence or absence of the mite on the body or inside the cell ^20^. All groups were replicated in three independent metal cages (30 bees per cage), that were set up on the same day and kept in an incubator under the same conditions described above ^55^. On the fifth day after emerging, two sample types for venom chemical analyses were obtained, for each bee: described above: (1) intact venom sac (source) and (2) body of the individual without the abdomen (head and thorax).

### Behavioural observations

To investigate whether grooming behaviours (*i.e.,* self-grooming and allogrooming) and the likely consequent application of venom to the bee’s body surface is influenced by *Varroa* infestation, we determined the frequency (e.g., number of events) of both grooming types by using the “all occurrences sampling” method ^57^. In addition, to evaluate the effectiveness of grooming in counteracting *Varroa* parasitism, the grooming behaviour was divided into three ethogram entries, as follows: (1) *cleaning body with legs or mandibles* + *shaking the abdomen*, (2) *removing mite using bee’s legs or mandibles*, and (3) *damaging the mite using the mandibles*. The frequency of each event entry was recorded. Thirty emerging bees from three *Varroa*-free colonies were mixed to prevent any family effects (ten bees from each colony) and placed in a metal cage as described above. To obtain adult workers of the same age parasitized during the adult stage (dispersal phase) and uninfested workers from the *Varroa*-free colonies, we used the method described above ^18^. The behavioural observations were repeated for three consecutive days (fifth to seventh day of bee age) during which infested and uninfested bee groups were compared. Self-grooming and allogrooming occurrences were counted every day, at three different time slots (9:00–9:30; 13:30–14:00; 18:00–18:30), with 30-min observation sessions, corresponding to a total of 1 h and 30 min per day per cage. The cages were kept in an incubator under the same conditions as described above ^55^. All treatments were replicated using three independent cages (30 bees per cage) and were set up at the same time.

### Toxicology bioassay

To evaluate the effect of bee venom on *Varroa* activity, five mites were placed in a 90-mm diameter Petri dish containing an absorbent paper 67 g/mq (APTACA SRL, Canelli, Italy) ^18^. Each mite was wetted with 1 µL of a water solution containing 0.2 µg of dried bee venom extract (CITEQ, Groningen, The Netherlands) or 1 µL of water solution (control group). Mites were obtained from brood cells capped in the preceding 15 h, following the procedure described by Nazzi et al. ^56^. A total of 90 mites from six *Varroa*-infested colonies were tested, corresponding to 45 mites per treatment (control *vs.* venom). Mite activity was observed under a stereo microscope every 15 min for the first hour, every 20 min for the second hour, and every 30 min for the next six hours. Mites were considered to be inactive when they showed no response to contact stimulus using a stick ^58^. This bioassay was replicated twice (180 mites in total). Both experiments were conducted under artificial light and at temperature of 32 ± 1 °C.

### Statistical analysis

We used a linear mixed model (LMM), followed by Tukey post-hoc tests to investigate differences in melittin concentration (mg/L) on the bodies of worker bees (freshly emerged bees, nurses and foragers) and drones. Colony was included as a random factor. Due to the absence of melittin on the bodies of freshly emerged worker bees and drones, statistical comparisons are reported only between nurse and forager worker bees.

An LMM, followed by Tukey post-hoc tests, were also used to examine the effect of treatment (control, sting-blocked, and mix) on melittin concentration on the bees’ bodies. The cage was included as a random factor. As melittin was absent in sting-blocked bees, comparisons were restricted to control bees housed in single and mixed cages. We further used LMMs, followed by Tukey post-hoc tests, to assess the effects of treatment (cell infestation, dispersal phase and *Varroa*-free) on melittin concentration in the bees’ bodies and venom glands, respectively. Additionally, an LMM was used to evaluate the effect of treatment on the proportion of melittin found on the body relative to the total melittin (body + venom sac). The cage was included as a random factor in all models.

To examine differences in grooming behaviour (self- and allogrooming) between infested and uninfested bees, we used generalized linear mixed models (GLMMs) with a negative binomial error structure. The experimental group was treated as a fixed factor, while time slot, day of experiment and cage were included as random effects. Additionally, to evaluate the effect of venom treatment (control vs. bee venom) on *Varroa* mite activity, we used a GLMM with a binomial error structure. Treatment, time of observation and their interaction were included as fixed effects and the dish was included as a random factor.

All analyses were performed in R v.3.5.2 ^59^. Mixed effects models were conducted using the R package *lme4* ^60^. Tukey tests were performed using the R package *multcomp* ^61^. Model assumptions were checked visually and found to meet expectations, including homogeneity of variance, normality of residuals, and linearity. Due to lack of homogeneity of variance and normality, melittin concentration was square root-transformed in all models.

## Results

### LC–MS/MS method validation

The correlation coefficient of the calibration lines (r^2^) showed values oscillating between 0.992 and 0.999. The linearity was, therefore, above the condition set for the validation of the method. The accuracy data provided by the recovery experiments obtained from six different replicates for the two concentrations tested ranged from 87.1 to 93.7% at the LOQ level and from 81.7 to 90.3% at the 10 × LOQ level (Table S3). The values obtained showed a good extraction capacity of the proposed method. Therefore, the method deemed suitable for the melittin analysis in the bee samples. The repeatability (RSDr) and reproducibility (RSDwR) of the tests showed values lower than 20%, thus showing good repeatability of the analytical method used. The chromatograms of the control and standards did not show the presence of interfering peaks, thus indicating a good selectivity of the method (Fig S1). The results obtained from the validation tests are consistent with the validation parameters of the SANTE/12,682 /2019 guidelines ^52^.

### Detection of melittin on the body of adult worker bees and drones

**Fig. 2:**
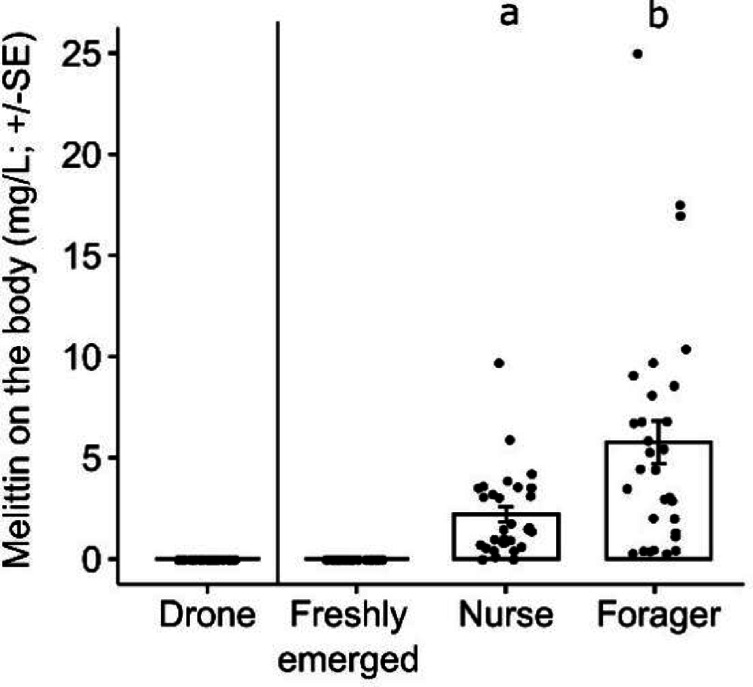
Amount (mg/L) of melittin (means ± SE). Melittin on the body of different type of bees: drones, freshly emerged bees, nurses, and foragers are shown. Different letters indicate significant differences among (LMM; Tukey post-hoc test; *P* ≤ 0.05).

We found a detectable amount of melittin only in nurse and forager bees (Fig. 2). No melittin was observed in drones and freshly emerged bees (Fig. 2). Interestingly, the amount of melittin detected on the bodies of nurses’ was significantly lower than that on foragers’ (LMM; Tukey post-hoc test; Z = 3.165, *P* = 0.001; Fig. 2).

### Origin of the melittin detected on the worker’s body

**Fig. 3:**
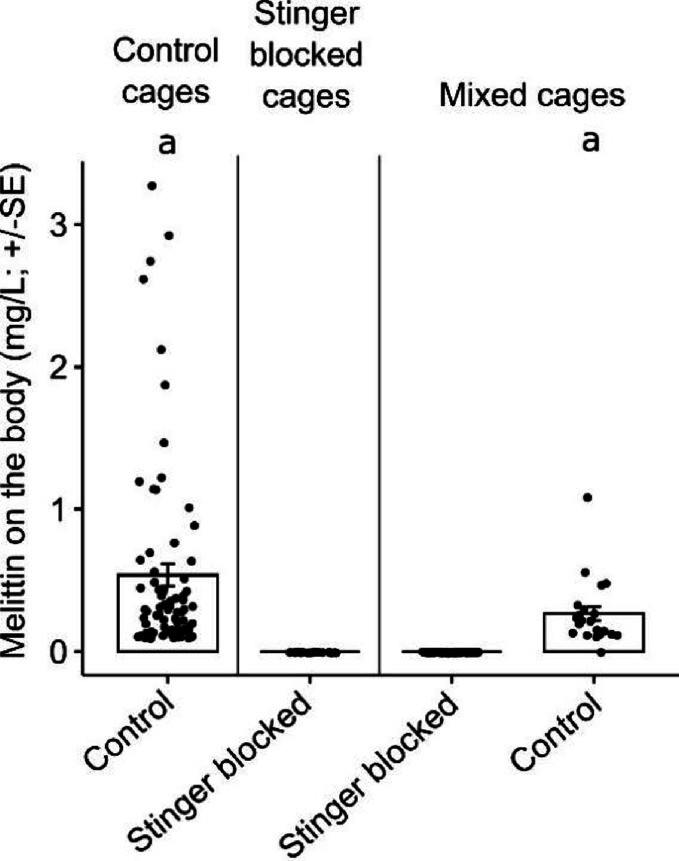
Amount (mg/L) of melittin (mean ± SE). Melittin on the body of control bees (unblocked stinger), bees with the stinger blocked, and a mixture of caged bees with and without the stinger blocked. Different letters indicate significant differences between groups (LMM; Tukey post-hoc test; *P* ≤ 0.05).

The experiment conducted to determine the amount of venom on the bodies of bees with blocked or unblocked stingers, whether housed individually or in groups, clearly demonstrated that the melittin present on workers’ bodies originates from the venom gland (Fig. 3). Specifically, no melittin was detected on the bodies of bees with blocked stingers, regardless of whether they were kept alone or alongside bees with unblocked stingers (Fig. 3).

### Detection of venom on the bodies of infested and uninfested workers and in the venom sac

**Fig. 4:**
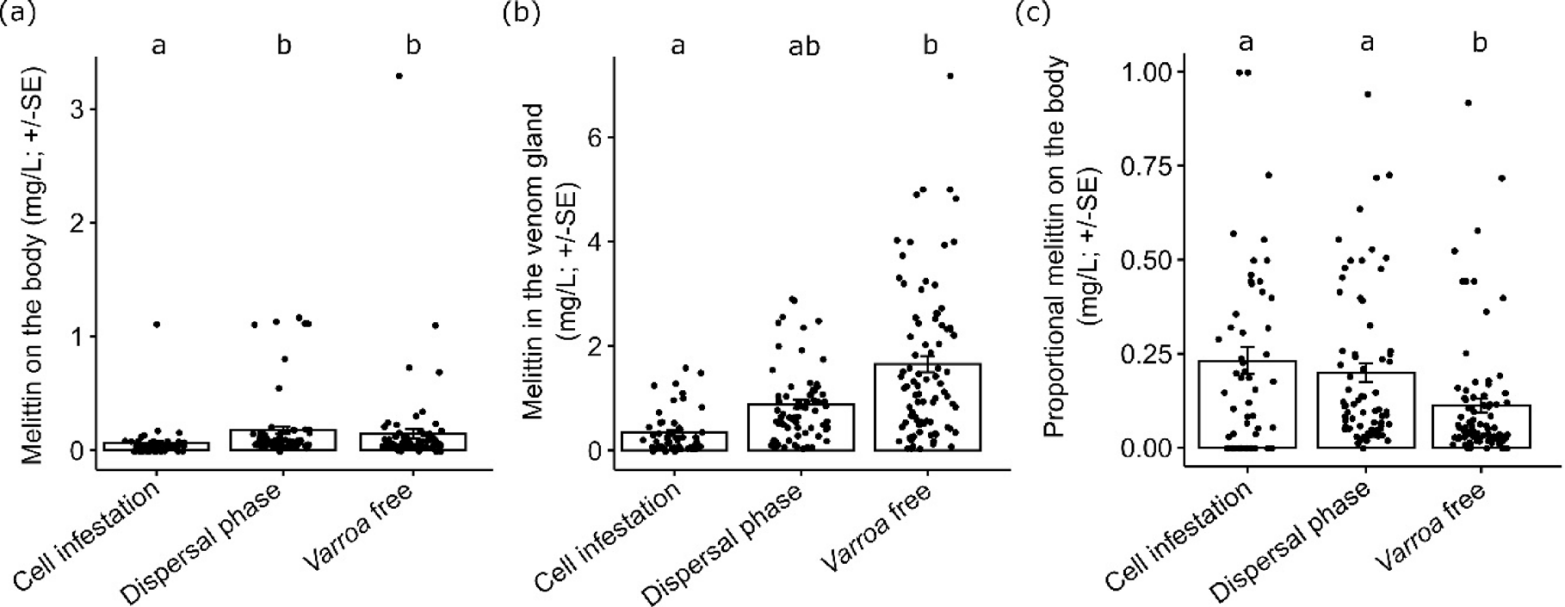
(**a**) Amount (mg/L) of melittin (mean ± SE) of workers infested inside the bee cells, infested workers in the dispersal phase, and control bees. (**b**) Melittin in the venom sac (mg/L) of workers infested inside the bee cells, infested workers in the dispersal phase, and control bees. (**c**) Proportional amount (mg/L) of melittin on the body **(**mean ± SE) of workers infested inside the bee cells, infested workers in the dispersal phase, and control bees. Different letters indicate significant differences between groups (LMM; Tukey post-hoc test; *P* ≤ 0.05).

The amount of melittin detected on the cuticle of worker bees parasitized during the pupal stage (cell infestation) was significantly lower than in those parasitized as adults (dispersal phase) (LMM; Tukey post hoc test; Z = 3.415, *P* = 0.001, Fig. 4a) and in uninfested control bees (LMM; Tukey post hoc test; Z = 2.275, *P* = 0.034, Fig. 4a). In contrast, there was no significant difference in the amount of melittin present on the bodies of adult-parasitized bees compared to the control bees (LMM; Tukey post hoc test; Z = 1.291, *P* = 0.196, Fig. 4a).

Furthermore, the amount of melittin detected in the venom sac was significantly lower in adult worker bees parasitized during the pupal stage than in control bees (LMM; Tukey post hoc test; Z = 2.442, *P* = 0.043, Fig. 4b). Similarly, in adult workers parasitized as adults, the amount of melittin in the venom sac was lower than in control bees (approximately half on average), although this difference was not statistically significant (LMM; Tukey post hoc test; Z = 1.434, *P* = 0.227, Fig. 4b). However, calculating the proportion of melittin found on the bodies of worker bees relative to total melittin amount (e.g., the sum of melittin present on the body and in the venom sac), showed that this proportion significantly increased in parasitized bees (both as adults and during the pupal stage) compared to unparasitized ones (LMM; Tukey post hoc test; Z = 3.308, *P* = 0.002; Z = 2.591, *P* = 0.014, respectively, Fig. 4c).

### Behavioural observations

**Fig. 5:**
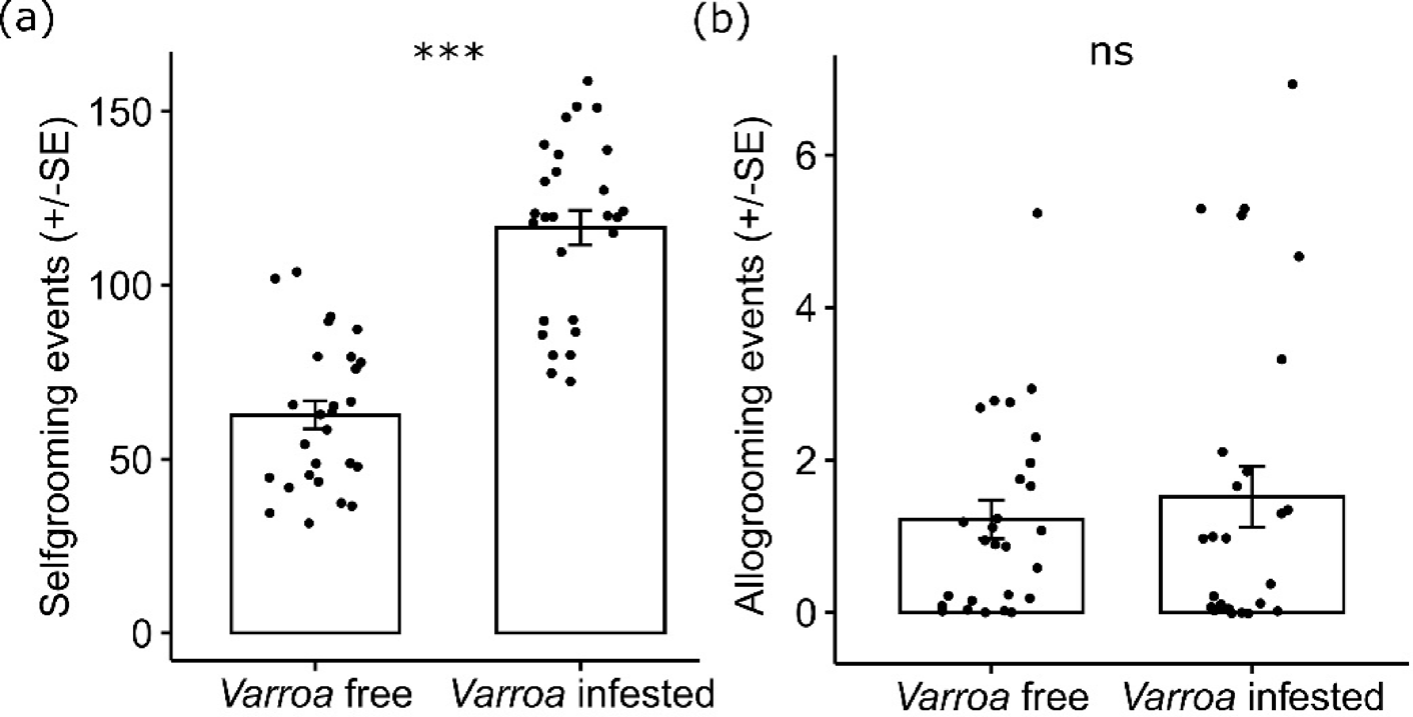
(**a**) Sel-fgrooming behaviour in *Varroa-*free *vs. Varroa*-infested bees (dispersal phase). Means and ± SE are shown. *** *P* < 0.001. (**b**) Allogrooming behaviour in *Varroa*-free *vs*. *Varroa*-infested bees (dispersal phase). Means and ± SE are shown. ns *P* > 0.05.

We observed a total of 4,841 self-grooming events in which bees were cleaning their body using the legs + shaking the abdomen. Out of those, 3,147 were observed in the dispersal phase group and 1,694 were in the uninfested group (65% *vs.* 35%). This difference was statistically significant (GLMM; χ^2^ = 27.973, *P* < 0.001; Fig. 5a). However, removing mites using the bees’s legs was observed only 12 times out of the 3,147 self-grooming events observed in the *Varroa*-infested group (0.38%). Damaging the mite using the bee’s mandibles did not occur in our study.

We observed a total of 74 allogrooming events by cleaning the nestmate’s body with their mandibles. Out of these, 41 were observed in the dispersal phase group and 33 were in the uninfested group (55% *vs.* 45%). This difference was not statistically significant (GLMM; χ^2^ = 0.090, *P* = 0.763; Fig. 5b). Removing mites from other bees using the mandibles and damaging them did not occur in our study.

### Toxicology bioassay

**Fig. 6:**
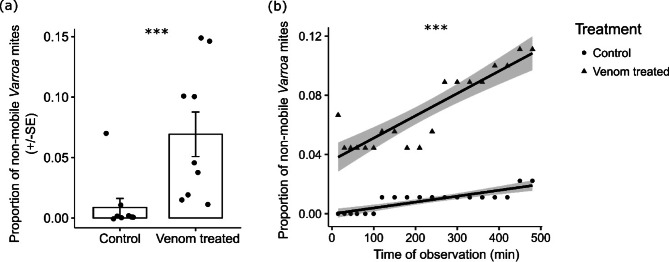
(**a**) Proportion of non-mobile (inactive) *Varroa* mites in a control water solution compared to those in the bee venom treatment. (**b**) Proportion of non-mobile (inactive) mites over time of observation. Means and ± SE are shown. Plotted line shows predicted relationship and the shaded area indicate the 95% confidence intervals. *** *P* < 0.001.

Exposure of the mites to honey bee venom resulted in a highly significant increase in the percentage of inactive mites compared to the control (approximately 7% *vs* 0.9%, respectively; GLMM; χ^2^ = 12.191, *P* < 0.001; Fig. 6a). The proportion of inactive mites also increased with the time of observation (GLMM; χ^2^ = 19.382, *P* < 0.001; Fig. 6b). The interaction between treatment and time was not statistically significant (GLMM; χ^2^ = 1.620, *P* = 0.203).

## Discussion

This study investigated the presence and origin of venom antimicrobial compounds on honey bees’ body as an external immune defence trait upon challenge by *V. destructor* together with venom effects on the parasite’s activity levels. We first confirmed the results of earlier studies ^43,44^ by showing that melittin, a venom compound, is absent on the body surface of drones and freshly emerged bees but it is present on nurse and forager workers. We then found that melittin was absent on the body of workers that had their stingers blocked, thus suggesting that venom may be transferred from the sting gland to the body surface, likely during self-grooming behaviour, and is not picked up from other sources in the hive environment. We also found that venom presence on the body surface was influenced by the presence of *Varroa*, with parasite presence leading, on one hand, to a proportional higher amount of melittin on the bee’s body surface relative to the total melittin (the sum of melittin present on the body and in the venom sac), and on the other hand, to a lower overall amount of melittin production. In addition, we found that *Varroa*-infested bees engaged in self-grooming behaviour more frequently than control bees. Although our study does not provide direct evidence for the distribution of venom on the bee body via self-grooming, the absence of melittin on bees with blocked stingers, combined with the proportionally higher amount of melittin on the surface of *Varroa*-infested bees, suggests that the increased self-grooming activity in these bees may facilitate the transfer of venom from the sting gland to the body surface. Lastly, we found that bee venom negatively influenced parasitic mite activity, thus indicating that venom deposition on the body surface of the bees may serve as an external immune defence trait against parasites and/or pathogens. As previously noted, bees with their stingers blocked, and therefore unable to release venom, did not show melittin on their bodies. This result remained consistent also when bees were reared in the same cage as control bees capable of releasing venom. These findings align with those reported by ^43^ and are further supported by our results that showed the absence of venom on the bodies of drones sampled from the hive and the presence of venom on the bodies of nurse and forager bees. Notably, drones are also groomed by workers, although this behaviour occurs less frequently than among workers ^62,63^. If allogrooming behaviour had been responsible for venom spread, then drones, newly emerged bees, or bees with blocked stingers would have had melittin on their bodies. In fact, our results suggest not only that venom on the body surface of worker bees is not just an artefact, but also that it is likely used only as an external immune defence for personal hygiene rather than for sanitation of other adult nest members. This is because venom is not spread via allogrooming to drones or bees with blocked stingers. In contrast, in ants, venom is used not only for personal sanitation via self-grooming e.g., ^64^ but also for the sanitation of developing brood ^27^ and adult nest members via allogrooming ^65^.

Interestingly, our research provides clear evidence that *Varroa* parasitisation leads to a lower amount of melittin in the honey bee venom. In fact, our results revealed that when bees were parasitized by *Varroa* during the pupal stage, the external immune system of worker bees could be weakened, as evidenced by the lower levels of melittin found on their cuticles and venom sacs compared to healthy bees. This negative effect was evident when bees were parasitized during the pupal stage but not when parasitism occurred in adults. To our knowledge, this result had never been reported before and we believe that it can be explained by the high energy cost of venom production in aculeate Hymenoptera ^22,66^ and the physiological impairments caused by *Varroa* on honey bees ^67,68^.

Assuming that bees cannot selectively choose which venom components to apply to their bodies and considering that parasitized bees use a greater proportion of the total melittin content from their venom sac compared to unparasitized bees, it is likely that parasitized worker bees spread larger amounts of venom to their bodies by engaging more frequently in venom bathing although this behaviour is highly costly, especially for diseased bees. However, this does not necessarily imply that the distribution of venom across the body represents a defence mechanism adopted by bees against *V. destructor*. In fact, the following question remains open: do bees use more venom (1) in response to *Varroa* attacks or (2) simply to maintain a minimum level of antimicrobial substances on their bodies for the control of opportunistic or pathogenic microbes? A finding supporting the second hypothesis was the significantly higher amounts of melittin found on the bodies of foragers compared to nurses in our trial and in literature ^33–36^. It is well known that foragers face higher pathogen exposure ^69^ and thus require additional protection compared to nurse bees. Therefore, it is debatable that bees seem capable of regulating the dosage of antimicrobial substances on their bodies according to their perceived level of protection needed. However, Baracchi and Turillazzi ^43^ found that nurses’ venom contains higher percentages of apamine and lower percentages of melittin compared to the venom of older nestmates (guards and foragers). Therefore, the difference we observed between the two cohorts of bees (nurses *vs.* foragers) likely resulted from variations in the composition of the venom distributed on the body rather than the quantity applied by the different bee cohorts.

As expected, a significant increase in self-grooming was observed in the group challenged with the ectoparasite *Varroa*. The behaviour of cleaning the body with legs and shaking the abdomen is likely involved in spreading of antimicrobial substances across the bee’s body. Like in previous studies conducted on ants ^21,26^, our results suggest that self-grooming in *A. mellifera* involves venom spread. However, our findings confirm the poor efficacy of self-grooming in combating *Varroa* in *A. mellifera*. Indeed, the observed percentage of removal of the parasite during self-grooming events was very low (only 0.38% of the cases), which was very similar to that observed by Peng et al. ^70^. Moreover, in accordance with Büchler et al. ^46^, we never observed a bee damaging the mite with its mandibles. In accordance with our findings on absence of melittin on the body of drones and blocked stinger bees, an increase in allogrooming was not observed in these bees. This is consistent with the results of previous studies that have demonstrated that this behaviour is rare and occurs mainly during the nursing period ^69,71^. In addition, we never observed the removal of parasites or bees damaging mites with their mandibles during allogrooming behaviour.

Our study also showed that bee venom has a detrimental effect on *Varroa* mites. However, the observed negative effect of the venom was relatively mild (approximately 7% of inactive mites) in comparison to other hive products such as raw propolis, which has an effect of 19–22% ^18^, or ethanol propolis extracts, which have an effect of 45–90% ^20,72^.

It could be assumed that venom on the bees’ body surface might provide protection against other honeybee parasites and/or pathogens besides *Varroa* ^73,74^. For example, it is known that some honey bee-associated viruses, namely Chronic bee paralysis virus and Israeli acute paralysis virus can be transmitted by topical application ^75^ and contact with a virus-contaminated environment or infected bees ^76–79^. Therefore, given the strong antiviral effect of melittin ^40^, venom on the bee body surface might also help against virus transmission and infection. In support of this hypothesis, previous work has shown that bee venom, as a dietary supplement, increased the expression levels of immune genes encoding the antimicrobial peptides Abaecin, Defensin 2 and Hymenoptaecin as well as stimulated the production of juvenile hormone and vitellogenin secretion, and decreased *Varroa* infestation level within the colony ^80^. Recently, ^81^ also showed an immunostimulatory as well as a beneficial role of venom in honeybees challenged by *Vairimorpha* (*Nosema*) *ceranae.* In addition, ^82^ found that melittin is also synthesized in the fat body, which is apparently tolerated by bee cells and most likely protects the bee from infection.

A hypothesis that would be interesting to test in future studies is whether the negative effect induced by the venom on mites could facilitate its detachment from the bee’s body during self-grooming. Given the differences in venom composition and venom deposition among *Apis* species ^44^, it would be interesting to study *venom bathing* behaviour in relation to parasite efficacy control, both in *A. mellifera* and *A. cerana,* with the latter being the original host of *Varroa destructor*. This deserves further attention as it might explain some of the variations in mite resistant traits between species, e.g., mite infertility ^83^. It would also be interesting to investigate whether bees distribute venom in varying amounts across different regions of their bodies in relation to the risk of exposure to parasites and/or pathogens. For example, the abdomen may require a higher concentration of antimicrobial substances, as it represents the region where *V. destructor* most frequently exerts its parasitic activity.

Another aspect that is important to consider is that the hive is a complex system where antimicrobial venom ^43^ coexists with other bioactive matrices such as propolis ^18^, honey ^84^, wax ^85^, pollen ^86^, and royal jelly ^87^. The combination of these matrices with their bioactive properties could result in a synergistic effect against pests and pathogens ^88,89^. A synergistic effect of different antimicrobial substances was found in the study of Brütsch et al. ^90^, who observed that wood ants can produce a potent antimicrobial cocktail by combining formic acid with tree resins.

In conclusion, our results support the hypothesis that, like ants, honeybees may rely on venom bathing behaviour as a form of external immunity, potentially counteracting opportunistic and pathogenic microorganisms. However, further experiments are required to validate this form of defence that can be compromised by *V. destructor*, whose parasitising activity on bee pupae leads to reduced melittin production in adult bees. This discovery reveals, to our knowledge, another previously unknown negative side effect caused by this pervasive honey bee ectoparasite.

## Supplementary Information

Below is the link to the electronic supplementary material.

Supplementary Material 1

Supplementary Material 2

## Data Availability

All data are provided within the supplementary information files.
