# Supplementary material for: Varroa destructor weakens the external immunity of western honey bees by impairing melittin production
Source: Sci Rep. 2025 Aug 20;15:30623. doi: 10.1038/s41598-025-13440-2 (PMC12368138; doi:10.1038/s41598-025-13440-2)
Supplement: Supplementary file 2 — Supplementary Material 2 [file 41598_2025_13440_MOESM2_ESM.docx]

**Supplementary material for**

***Varroa destructor* weakens the external immunity of western honey bees by impairing melittin production**

Michelina Pusceddu^1,2*^, Simon Tragust^3*^, Panagiotis Theodorou^3^, Irene Ciabattini Bolla^1^, Jorge Sánchez Navarro^1^, Francesco Corrias^4^, Alessandro Atzei^4^, Alberto Angioni^4^, Ignazio Floris^1,2^, Alberto Satta^1,2^

1 Department of Agricultural Sciences, Section of Plant Pathology and Entomology, University of Sassari, Sassari, Italy, 2 National Biodiversity Future Center (NBFC), Palermo, Italy, 3 General Zoology, Institute of Biology, Martin Luther University Halle-Wittenberg, Halle (Saale), Germany, 4 Department of Life and Environmental Sciences, University of Cagliari, Cagliari, Italy

**Corresponding authors:* [mpusceddu@uniss.it](mailto:mpusceddu@uniss.it) (MP); [simon.tragust@zoologie.uni-halle.de](mailto:simon.tragust@zoologie.uni-halle.de) (ST)

**Table S1** Characteristic m/z ions during the ionization of melittin (MW 2646.75) operating in ESI mode.

| Ion | m/z* |
| --- | --- |
| MH^+2^ | 1423.88 |
| MH^+3^ | 949.59 |
| MH^+4^ | 712.44 |
| MH^+5^ | 570.16 |
| MH^+6^ | 475.30 |

*m/z: mass to charge ratio

ESI: electrospray ionization

**Table S2** Optimized MS/MS conditions for the target analyte in positive ESI mode using the LC-MS/MS method in the MRM* setting.

| Precursor ion (m/z) | Product ion (m/z) | Fragmentor (v) | Collision energy (v) |
| --- | --- | --- | --- |
| 713 | 143 | 150 | 42 |
|  | 86 | 150 | 58 |

*MRM: multiple reaction monitoring

**Table S3** Linear range, linear equation, correlation coefficient, LOQ, apparent recovery (%), precision (RSDr, and RSDwr; n=), uncertainty (U) of melittin using the LC-MS/MS method.

| Analyte | Linearity  (µg mL^-1^) | Linear regression equation | R^2^ ± RSD% | LOQ  (µg mL^-1^) | Apparent recovery (%) | | RSDr% | RSDwr% | U |
| --- | --- | --- | --- | --- | --- | --- | --- | --- | --- |
|  |  |  |  |  | LOQ | 10 x LOQ | LOQ | LOQ |  |
| Melittin | 0.1 – 5.0 | y=66954.8x + 31352.9 | 0.997 ± 0.33 | 0.1 | 87.1±9.4 | 81.7±13.5 | 12.6 | 17.7 | 21.2 |
|  |  |  |  |  | 93.7±2.4 | 90.3±3.3 | 10.5 | 15.3 | 14.7 |

Linearity: the calibration curve linearity was measured between 0.1 and 5.0 µg mL^-1^

LOQ: limit of quantification.

RSDr%: relative standard deviation intraday repeatability

RSDwr: relative standard deviation interday repeatability

Apparent recovery (%): average percent of ‘measured concentration/spiked amount’.

U: expanded uncertainty, U = k x u′; u′ = 𝑢′(𝑏𝑖𝑎𝑠)^2^ + 𝑢′(𝑝𝑟𝑒𝑐𝑖𝑠𝑖𝑜𝑛)^2^, k = 2 (coverage factor), to accomplish a level of confidence of 95%.

**Figure S1** Chromatograms of melittin in positive ESI mode using the LC-MS/MS method in the MRM setting.


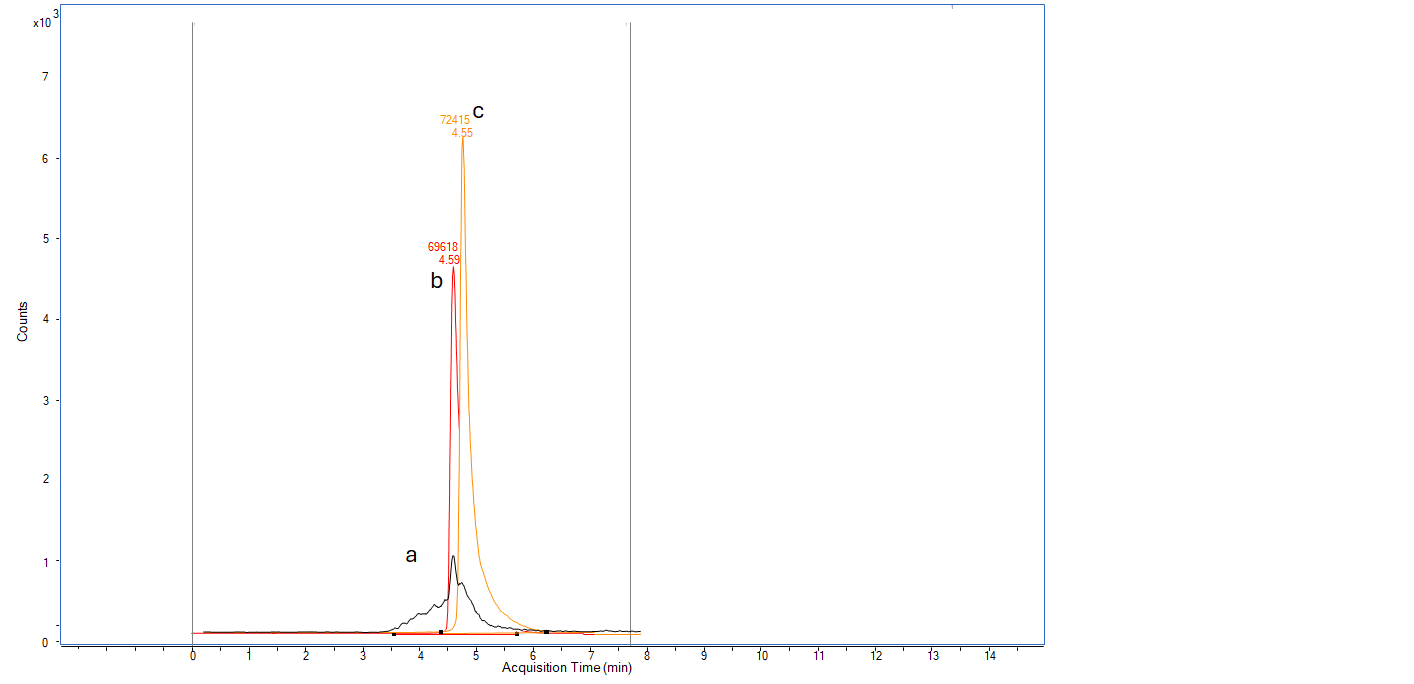


a) blank control, b) std at 250 µg L^-1^, and c) melittin residue on worker bees.
